# Challenges and lessons learned for REDD+ finance and its governance

**DOI:** 10.1186/s13021-023-00228-y

**Published:** 2023-05-18

**Authors:** Kanako Morita, Ken’ichi Matsumoto

**Affiliations:** 1 Center for Biodiversity and Climate Change, Forestry and Forest Products Research Institute, Ibaraki, Japan; 2 United Nations University Institute for the Advanced Study of Sustainability (UNU-IAS), Tokyo, Japan; 3 Faculty of Economics, Toyo University, Tokyo, Japan

**Keywords:** REDD+, Finance, Governance, Climate change mitigation, UNFCCC

## Abstract

Discussion on reducing emissions from deforestation in developing countries began at the United Nations Framework Convention on Climate Change (UNFCCC) Conference of the Parties in 2005, and the agenda for “reducing emissions from deforestation and forest degradation, and the role of conservation, sustainable management of forests and enhancement of forest carbon stocks in developing countries (REDD+)” was introduced under the UNFCCC. The REDD+ framework was developed with the expectation that it would significantly contribute to climate change mitigation at a relatively low cost and produce benefits for both developed and developing countries. Finance is a key element of REDD+ implementation, and many financial sources, approaches, and mechanisms have supported REDD+-related activities in various developing countries. However, the comprehensive challenges and lessons learned for REDD+ finance and its governance have not been fully explored. This paper reviews the relevant literature to understand the challenges for REDD+ finance and its governance in two areas—(1) REDD+ finance aligned with the UNFCCC and (2) REDD+-related finance outside the UNFCCC—which have developed differently and have different implications. This paper first identifies the six key elements of REDD+ finance and its governance across the two fields, and then reviews the related challenges and lessons learned with respect to public and private finance. The challenges for REDD+ finance and its governance aligned with the UNFCCC include enhancing the performance of REDD+ finance using mainly public finance, such as results-based finance and the jurisdictional approach. In contrast, the challenges regarding REDD+-related finance outside the UNFCCC include enhancing the engagement of the private sector in REDD+ finance, mainly targeting the project level, and the relationship between voluntary carbon markets and other investment and finance mechanisms. This paper also identifies the common challenges across REDD+ finance and its governance in the two fields. These challenges include the need to enhance linkages between REDD+ and other objectives, such as carbon neutrality/net-zero, deforestation-free supply chains, and nature-based solutions, as well as the need to develop learning systems for REDD+ finance.

## Background

The agriculture, forestry, land use, and land use change sector is a significant net source of greenhouse gas (GHG) emissions and contributed approximately 23% of anthropogenic emissions of carbon dioxide (CO_2_), methane, and nitrous oxide, combined as CO_2_ equivalents, during 2007–2016 [1]. Forest conservation and avoided deforestation and degradation are immediate alternatives for climate change mitigation [2]. The discussion on reducing emissions from deforestation in developing countries began in 2005 at the United Nations Framework Convention on Climate Change (UNFCCC) Conference of the Parties (COP), COP11, under the proposition by Papua New Guinea and Costa Rica [3]. Since then, the agenda for “reducing emissions from deforestation and forest degradation, and the role of conservation, sustainable management of forests and enhancement of forest carbon stocks in developing countries (REDD+)” has been introduced under the UNFCCC. The REDD+ framework was developed with the expectation that it could significantly contribute to climate change mitigation at a relatively low cost and benefit both developed and developing countries [4–6]. At the UNFCCC COP13 in 2007, REDD+ was incorporated into the Bali Action Plan, which is a comprehensive process to enable the full, effective, and sustained implementation of the Convention through long-term cooperative action now, up to, and beyond 2012 (Decision 1/CP.13). At the UNFCCC COP16 in 2011, the Cancun Agreements were adopted (Decision 1/CP.16), which moved REDD+ firmly forward as a key component of the post-2012 international climate change regime by describing its key elements, including finance, and operationalizing its initial phase [7].

The Warsaw Framework for REDD+—which forms its basic rules, including results-based finance; coordination of support; national forest monitoring systems; safeguards (which ensure that REDD+ does not harm the environment and local people) [8]; forest reference emission levels and/or forest reference levels (which serve as benchmarks for assessing each country’s performance in the implementation of REDD+ activities) [9]; measurement, reporting, and verification (MRV); and addressing the drivers of deforestation and forest degradation—was adopted at the UNFCCC COP19 in 2013 (Decisions 9–15/CP.19). In 2015, the UNFCCC COP21 adopted the Paris Agreement, aiming to strengthen the global response to the threat of climate change from the viewpoints of sustainable development and poverty eradication (Article 2). Forests play an important role in achieving the goals of the Paris Agreement, and the REDD+ framework is recognized in Article 5 of the Agreement [10]. Regarding nationally determined contributions (NDCs), which are the efforts of each country to reduce national emissions and adapt to climate change impacts under the UNFCCC [11], REDD+ is a potential mitigation alternative that can be included in these contributions [12]. Forest-related carbon trading is eligible under NDCs through internationally transferred mitigation outcomes (Articles 6.2 and 6.3 of the Paris Agreement) [12]. If well implemented, REDD+ can significantly contribute to climate change mitigation and yield other co-benefits, including climate change adaptation, biodiversity conservation, and poverty reduction [13–15].

Finance, including bilateral and multilateral, public and private, and international and domestic, is pivotal to incentivizing and implementing REDD+ activities using different approaches, such as results-based finance and voluntary carbon markets. Although finance is a key element of REDD+ implementation, the characteristics of REDD+ finance and its governance, as well as the challenges and lessons learned to build effective REDD+ finance, have not been fully reviewed. Existing studies related to REDD+ finance have focused on particular funding sources or finance for specific programs and projects [16–21]. There is a lack of reviews on understanding the challenges for comprehensive REDD+ finance and its governance, which are important for improving REDD+ finance and the broader environmental finance.

This review aimed to identify the challenges for REDD+ finance and its governance in two areas—(1) REDD+ finance aligned with the UNFCCC and (2) REDD+-related finance outside the UNFCCC—using the relevant literature. These challenges and experiences of REDD+ can be used for the current discussion of finance for nature-based solutions including REDD+, as presented in sections “REDD+-related finance outside the UNFCCC” and “Utilizing challenges and lessons learned to improve REDD+ finance and broader environmental finance”. In this paper, we use the term “REDD+ ” broadly, not just limited to REDD+ aligned with the UNFCCC but also including REDD+ outside the UNFCCC. This is because this term has been used broadly in practical and academic discussions. Furthermore, we define the governance of REDD+ finance as a set of processes, including interactions between institutions and actors in both the public and private sectors, aiming to promote REDD+ implementation through financing.

## Main text

### Background of REDD+ finance

#### REDD+ finance and its governance

REDD+ is one of a few items on the UNFCCC negotiation agenda that has been supported by both developed and developing countries [22]. In theory, REDD+ can provide benefits to both developed and developing countries: developed countries can reduce emissions at a relatively low cost by supporting REDD+, and developing countries can receive financial incentives to reduce emissions from the forest sector [4–6].

The Cancun Agreements clearly stated the need for providing adequate and predictable support for REDD+ in developing countries, including financial resources and technical and technological support, and requested the exploration of financing options for the full implementation of results-based activities of REDD+ (Decision 1/CP.16). Since then, results-based finance for REDD+ has been actively discussed. The UNFCCC COP18 in 2012 decided to undertake a work program on results-based finance in 2013 to ensure the full implementation of REDD+ activities and addressed options including (1) ways and means to transfer payments for results-based actions, (2) ways to incentivize non-carbon benefits, and (3) ways to improve the coordination of results-based finance [23]. This work program concluded at the UNFCCC COP19 in 2013, in Decision 9/CP.19, which provided guidance to the financing and support for the REDD+ activity implementation for parties and entities financing such activities [23]. In Decision 9/CP.19, the COP (1) reaffirmed that results-based finance can originate from various sources, including public and private, bilateral and multilateral, and alternative sources; (2) encouraged financing entities, including the Green Climate Fund (GCF) in a key role, to collectively channel adequate and predictable results-based finance in a fair and balanced manner and to work with a view to increase the number of countries that are in a position to obtain and receive payments for results-based actions; (3) encouraged financing entities to continue to provide financial resources to alternative policy approaches, such as joint mitigation and adaptation approaches, for the integral and sustainable management of forests; (4) recognized the importance of incentivizing non-carbon benefits for the long-term sustainability of the implementation of REDD+ activities; (5) decided to establish an information hub on the REDD+ Web Platform to publish information on the results and corresponding results-based payments; and (6) requested the Standing Committee on Finance (SCF) to consider issue of financing for forests in its work on coherence and coordination [23].

Currently, REDD+ activities are financed through various sources (e.g., multilateral and bilateral, public and private, and international and domestic), which are linked to different finance approaches and mechanisms (e.g., results-based finance and voluntary carbon markets) [24]. Under the UNFCCC, REDD+ is implemented in the following phased approach: phase 1 is readiness, phase 2 is implementation, and phase 3 is results-based payment. The results and performance of REDD+ are largely measured in terms of emission reductions calculated on the basis of forest reference (emission) levels, and these emission reductions are compensated for by results-based payments in phase 3 [25]. To date, at least USD 5.4 billion funding has been committed for REDD+ in the three phases through multiple development financial institutions [26]. The main source for the support in the three phases is public finance, and most of the REDD+ finance is spent in the readiness phase [27, 28]. Regarding private finance, the current private sector contribution to REDD+ is mainly through voluntary carbon markets and the project-scale payments for carbon offsets/units [27]. Currently, there is no adequate, predictable, and sustainable source of finance for REDD+ [14, 15, 27, 29].

#### Overview of REDD+ finance implementation

To better understand the overall picture of REDD+ finance, this section summarizes the current status of REDD+ finance aligned with and outside the UNFCCC by highlighting public and private finance sources, which are the main financial sources in the respective areas.

##### Multilateral and bilateral public finance aligned with the UNFCCC

**Fig. 1:**
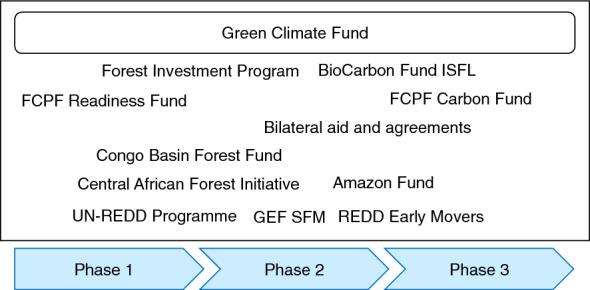
Public funds and programs supporting REDD+ implementation (created based on GCF [32]). *FCPF, Forest Carbon Partnership Facility; ISFL, Initiative for Sustainable Forest Landscapes; GEF SFM, Global Environment Facility Sustainable Forest Management

The REDD+ activities aligned with the UNFCCC are mainly supported by multilateral and bilateral public finance [30, 31]. Public finance for REDD+ comprises a wide range of public funds and programs that support different phases of REDD+ implementation, as shown in Fig. 1 [24, 32]. The GCF, which is the only stand-alone multilateral financing entity mandated to serve the UNFCCC [33], supports REDD+ activities in all phases. In the GCF’s first replenishment (2020–2023), USD 10 billion in pledges was raised for climate change mitigation and adaptation, including REDD+ [34]. The GCF is recognized by the UNFCCC as a key funding instrument to finance REDD+ results-based payments, as described in Decision 9/CP.19 [32]. In October 2017, the GCF Board approved a request of USD 500 million for proposals under a pilot program for REDD+’s results-based payments [35].

The Global Environment Facility (GEF)—which serves as a financial mechanism for international environmental conventions, including the UNFCCC—has also supported REDD+ through its program on sustainable forest management (SFM) [36]. The support has been mainly provided for phase 2 [36], and currently, the SFM program does not focus on REDD+, although it is complementary to REDD+ activities [37].

**Fig. 2:**
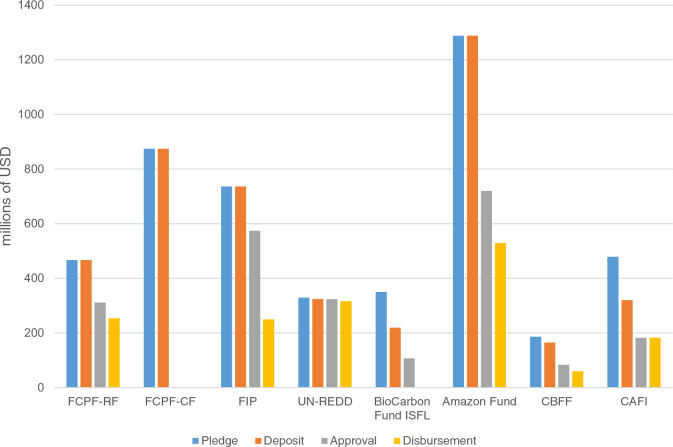
Multilateral funds for REDD+. *Based on the data obtained from the Climate Funds Update [38]. The data are up to date as of December 2020

In addition to the GCF and GEF, which serve the UNFCCC, there are other types of multilateral funds and programs for REDD+, including those managed by international organizations, such as the World Bank’s Forest Carbon Partnership Facility (FCPF)—Readiness Fund (RF) and FCPF-Carbon Fund (CF), the World Bank’s Forest Investment Program (FIP), the World Bank’s BioCarbon Fund Initiative for Sustainable Forest Landscapes (ISFL), and the UN-REDD programme, which is a UN collaborative program on REDD+ led by the Food and Agriculture Organization of the United Nations, the United Nations Development Programme, and the United Nations Environment Programme. There are also multilateral funds and programs for specific countries and regions, such as the Amazon Fund (for the Brazilian Amazon), the Congo Basin Forest Fund (CBFF; for Congo Basin), the Central African Forest Initiative (CAFI; for Central African countries), and REDD Early Movers (in Brazil, Ecuador, and Colombia). The FCPF-RF, FIP, UN-REDD programme, CBFF, and CAFI support mainly phase 1 and phase 2, while the FCPF-CF, BioCarbon Fund ISFL, Amazon Fund, and REDD Early Movers mainly support phase 2 and phase 3 (Fig. 2).

Since 2008, USD 5.2 billion has been pledged to multilateral climate funds that support REDD+ efforts, and cumulatively, USD 2.8 billion has been approved for dedicated REDD+ activities [39]. The Amazon Fund is the largest dedicated REDD+ fund, with USD 720 million approved for 103 projects in Brazil and the Amazon biome [39].

Bilateral aid and bilateral agreements are also a part of REDD+ public finance. Bilateral finance is implemented on the basis of the national official development assistance criteria. Its funding is allocated through national authorities, and various bilateral finance approaches for REDD+ have been developed [30].

The European Union, Germany, Japan, Norway, the United Kingdom, and the United States are the major contributors to REDD+ implementation [27, 40]. The support from these donor countries mainly focuses on the various activities of phase 2 and phase 3—such as delivering technical assistance to REDD+ countries, building capacity for MRV, and strengthening forest governance—with each donor country having its own priorities [27, 40].

In addition, there has been a long-standing interest in the potential to use market-based mechanisms to support REDD+ programs [39]. There are two types of carbon markets: compliance carbon markets (in which regulated entities obtain and surrender emissions allowances or offsets to meet regulatory emissions reduction targets) and voluntary carbon markets [26]. The links between voluntary carbon markets and REDD+ are important to be identified for mobilizing private finance for REDD+ (see sections “Overview of REDD+ finance implementation” and “REDD+-related finance outside the UNFCCC”). Other possible public finance sources for REDD+ include the use of state budgets and fiscal measures for REDD+, such as taxes and subsidies [24].

##### Mainly private finance outside the UNFCCC

Regarding the financing gaps, according to the Sixth Assessment Working Group III report of the Intergovernmental Panel on Climate Change, to meet the needs for rapid deployment of climate change mitigation options, global mitigation investments need to increase by a factor of 3–6 [15]. The gaps are wide across all sectors and a major challenge for relatively developing countries and for specific sectors like agriculture, forestry, and other land use and specific groups with limited access to, and high costs of, climate finance [15]. Because public sector resources cannot sufficiently meet the needs of REDD+ finance, supplementing public finance with private sector investments will become critical for mobilizing adequate finance for REDD+ [26, 27]. Private financial investments mainly support projects for REDD+ activities outside the UNFCCC, and private finance support is currently limited mostly to voluntary carbon markets [41]. Forestry and land use is the leading category in terms of volumes transacted in voluntary carbon markets [42] (see section "REDD+-related finance outside the UNFCCC”). Other potential private finance sources for REDD+ include green bonds, private foundations, and cooperative social responsibility [24]. In addition, blended finance schemes that combine public and private finance may also contribute to mobilizing private finance for REDD+ [32] (see section “REDD+-related finance outside the UNFCCC”).

### Framework for the literature review of REDD+ finance

REDD+ finance and its governance are complex and fragmented, containing diverse finance sources, mechanisms, and many institutions and actors related to REDD+ activities within and outside the UNFCCC. REDD+ finance and its governance have been discussed in both the practical literature [24, 27, 39, 43] and academic literature [30, 41, 44, 45]. The practical literature mainly maps different REDD+ finance options and shows the updated state and challenges for the finance. The academic literature includes case studies of specific REDD+ finance instruments and activities in recipient countries, or analyzes of the relationship between finance and other technical/institutional elements of REDD+. However, only a few academic studies have analyzed REDD+ finance and its governance, especially with regard to their recent status and challenges.

**Fig. 3:**
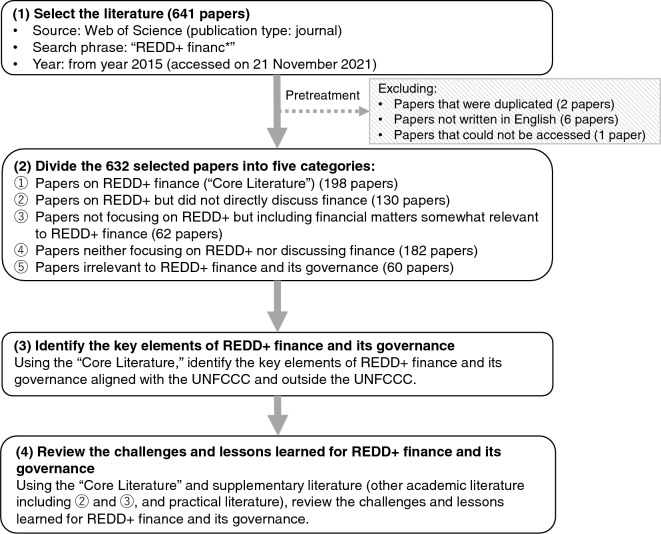
Framework for literature review of REDD+ finance and its governance

In this paper, we mainly review the academic literature related to REDD+ finance and its governance in two areas: (1) REDD+ finance aligned with the UNFCCC and (2) REDD+-related finance outside the UNFCCC. Figure 3 summarizes the flow of this literature review.

We collected the academic literature through the Web of Science (accessed on November 21, 2021) and selected the papers published in and after 2015, which is the year the Paris Agreement was adopted. Although REDD+ has been formally stated in the UNFCCC decision since 2007, the issues in the REDD+ finance debate have changed along with the discussion of the institutional design of REDD+ under the UNFCCC. Because the primary purpose of this paper is to identify the current challenges for REDD+ finance and its governance, we used recent papers since 2015. We selected the papers (publication type: journal) using the keyword “REDD+ financ*.” From the selected 641 papers, we excluded 2 papers that were duplicated, 6 papers not written in English, and 1 paper that could not be accessed. We then divided the remaining 632 papers into five categories: (1) papers on REDD+ finance (198 papers), which we called “core literature”; (2) papers on REDD+ that did not directly discuss finance (130 papers); (3) papers that did not focus on REDD+, although they included financial matters that were somewhat relevant to REDD+ finance (62 papers); (4) papers that did not focus on REDD+ and did not discuss finance (182 papers); and (5) papers that were irrelevant to REDD+ finance and its governance (60 papers).

In this review, we first identified the key elements of REDD+ finance and its governance aligned with the UNFCCC and outside the UNFCCC using the 198 papers on REDD+ finance as the core literature. Then, on the basis of the key elements of REDD+ finance and its governance, we reviewed the challenges and lessons learned, using both the core literature and supplementary literature. The supplementary literature involved other academic literature including papers categorized in (2) and (3), as well as the practical literature including reports of researchers, international organizations, and initiatives, mainly to supplement discussion that has limited literature such as that on private finance for REDD+.

### Review of challenges and lessons learned for REDD+ finance and its governance

#### Key elements of REDD+ finance and its governance

**Fig. 4:**
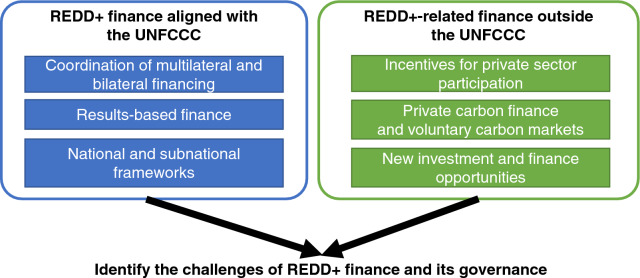
Key elements of REDD+ finance and its governance

This section summarizes the key elements of REDD+ finance and its governance identified using the core literature on REDD+ finance published since 2015 (Fig. 4). Regarding finance for REDD+ aligned with the UNFCCC, the three key elements are (1) coordination of multilateral and bilateral financing; (2) results-based finance, which is an important element in the phased approach; and (3) national and subnational frameworks, including benefit-sharing and safeguards. Regarding REDD+-related finance outside the UNFCCC, the three key elements are (4) incentives for private sector participation; (5) private carbon finance and voluntary carbon markets; and (6) new investment and finance opportunities. Based on the above key elements, we reviewed the challenges and lessons learned for REDD+ finance and its governance.

#### REDD+ finance aligned with the UNFCCC

##### Coordination of multilateral and bilateral financing

One of the main issues related to REDD+ finance and its governance is fragmentation and complexity. The institutional fragmentation of REDD+ finance and international public finance mechanisms, which are the main sources of REDD+, are well covered in the literature [16, 30, 46, 47]. Kim et al. [46] explained that the fragmentation and complexity of international REDD+ cooperation have emerged because each country or multilateral organization has supported REDD+ implementation in developing countries independently.

REDD+ activities aligned with the UNFCCC are mainly supported by multilateral and bilateral public finance [30]. However, the multilateral regime under the UNFCCC still lacks a cohesive mechanism to govern the sources, terms, and levels of REDD+ finance [48].

UNFCCC Decision 1/CP.21 identifies “the coordination of support from, inter alia, public and private, bilateral and multilateral sources, such as the Green Climate Fund, and alternative sources in accordance with relevant decisions by the Conference of the Parties.” Among the various multilateral and bilateral public finance sources, policymakers and researchers expect the GCF to play a central role in supporting REDD+ and in increasing transparency, coordination, and funds for REDD+, because there is no designated entity that can manage the funds [16, 30]. Although the GCF is an operating entity of the financial mechanism of the UNFCCC [49], it has established its own structures and policies to gather and deliver support for REDD+ from the readiness phase to the full implementation phase [16]. According to their review of the rules elaborated by the GCF for REDD+, Recio [16] showed that the GCF has completed, adjusted, and supplemented the UNFCCC COP guidelines. However, because the GCF established its rules independently from the UNFCCC guidance on REDD+, this may increase fragmentation, and more complex and specific requirements will mean higher entry costs and less incentive to participate [16]. Therefore, the GCF cannot fully address the challenges of fragmentation and complexity.

Within the UNFCCC, the SCF recommended to the COP that parties “ensure policy coherence, coherence of financing instruments and financial incentives and multisectoral coordination to address the drivers of deforestation and forest degradation, and promote sustainable management of forests” [50]. The SCF’s role is to provide a platform for a wide range of climate finance stakeholders to exchange information and promote linkages and coherence in mobilizing and delivering climate finance [51]. However, the SCF can only address fragmentation regarding the extent to which all countries agree on a voluntary mode of coordination [30].

Kim et al. [47] reported that the fragmentation of REDD+ financing explains both the inefficiency of aid allocation, which can be improved by aligned supports, and the diversity of REDD+ finance, which is linked to accessibility to support. Their analysis showed that there was relatively less fragmentation of REDD+ finance among recipient countries compared to that of general official development assistance because most of the supported finance came from a few major donors, such as Norway and Japan. By analyzing the ratio of fragmentation based on the different categories of donor–recipient relationships in the global REDD+ finance system (donor–recipient relationships that are significant for both donors and recipient countries, those significant only for donors, those significant only for recipients, and the relationships that are significant for neither donors nor recipients), they showed that compared to multilateral REDD+ finance, the fragmentation of bilateral REDD+ finance was higher because developed country donors tended to concentrate support to recipient countries that could favorably realize their own motivations [47].

In addition to the challenges related to the coordination of multilateral and bilateral REDD+ finance, other issues of fragmentation and complexity regarding REDD+ finance include multiple results-based finance approaches and different finance allocations to national and/or subnational programs or REDD+ projects [52, 53], as discussed in sections “REDD+ finance aligned with the UNFCCC” and “REDD+-related finance outside the UNFCCC”, and fragmented institutions on both the supply and demand sides in REDD+ finance [30], including complex donor–recipient relationships [54] and complex domestic institutional issues in recipient countries, as shown in section “REDD+ finance aligned with the UNFCCC”.

To address the multilevel institutional coordination of REDD+ involving multiple stakeholders, although the studies are limited, Gupta et al. [55] analyzed how the voluntary, multi-stakeholder REDD+ Partnership, which brings together state and non-state actors from global to local scales, has supported the management of fragmentation in international REDD+ financing mechanisms. They showed that the REDD+ Partnership has partially succeeded in achieving such procedural objectives, but that this has not resulted in the scaling up of REDD+ action and finance. These findings demonstrate the importance of coordination at the international level. Kim et al. [47] suggested that REDD+ finance coordination needs an information-sharing and monitoring system at the international level that collects information on ongoing REDD+ cooperation, the REDD+ finance commitments and disbursements, and the support needs of REDD+ countries.

##### Results-based finance

As shown in section “REDD+ finance and its governance”, REDD+ uses a phased approach. In the full implementation phase, it provides an ex-post reward for the results of REDD+ actions, known as results-based payment/finance, to incentivize a REDD+ country (the recipient) to undertake REDD+ actions [56]. The Warsaw Framework for REDD+ defines the international criteria for developing countries to reduce emissions and enhance forest carbon stocks and, in return, enables results-based finance for the measured GHG reductions and removals [41]. Results-based finance approaches vary depending on whether the finance is non-market-based, such as international public funds, or market-based, which is obtained by selling emission reduction units/carbon credits into global carbon markets [52, 53]. Finance for REDD+ results can flow to the jurisdictional (national and/or subnational) programs that are consistent with the UNFCCC, as well as to REDD+ projects that are implemented mainly outside the UNFCCC [53].

As indicated in UNFCCC Decision 9/CP.19, the GCF has been expected to play a key role in collectively channeling adequate and predictable results-based finance in a fair and balanced manner. REDD+ results-based finance is provided for REDD+ actions that are fully measured, reported, and verified and have met all UNFCCC requirements [16]. As described in section “Overview of REDD+ finance implementation”, in accordance with the UNFCCC, the GCF began to pilot REDD+ results-based payments in 2017. Results-based finance has also been piloted by the FCPF and the BioCarbon Fund ISFL, as well as other programs such as Norway’s International Climate and Forest Initiative and Germany’s REDD Early Movers Program [41]. Multilateral and bilateral donors have different principles, although they endeavor to be consistent with the vague requirements of UNFCCC decisions related to results-based finance [30, 56]. Currently, REDD+ results-based finance has largely been through donor government payments to developing countries, although the private sector has recently increased its interest in purchasing nature-based carbon offsets [57].

The challenges and lessons learned for existing REDD+ results-based finance, such as the GCF and the Amazon Fund, have been extensively analyzed [16, 17, 58, 59]. Christen et al. [59] examined the early lessons from the GCF’s pilot program for REDD+ results-based payments, as well as its assessment and verification procedures. They showed that the GCF’s pilot program placed a significant burden on countries’ abilities to access results-based finance because it required them to demonstrate compliance with its interim safeguards in REDD+ results-based finance. Recio [16] indicated that the GCF’s weakness lies in its limited ability to gather additional funds for REDD+. This shortage of funding cannot be addressed independently because donors cannot earmark their contributions for specific funding windows, activities, or countries [16]. van der Hoff et al. [58] described the issues for results-based finance in the Amazon Fund in terms of discursive conflicts between the recipient and donor countries regarding what constitutes “results” or “performance.” There are different interpretations of the temporal (i.e., past or future) and epistemological (i.e., how to measure) aspects of the results for which these payments are intended [58]. For example, Correa et al. [17] showed that the resource distribution from the Amazon Fund lacks a clear strategy that could maximize the Fund’s results in terms of reducing deforestation. They concluded that the Amazon Fund and other results-based finance programs need to evolve over time to develop a more targeted funding strategy to maximize their long-term impact on reducing emissions from deforestation.

Results-based finance has been at the core of the discussion on REDD+, in relation to financial uncertainty and the performance element in REDD+ [60]. Studies on the performance of REDD+ and results-based finance schemes have increased recently [17, 25, 61, 62]. Although results-based finance for REDD+ has become an important instrument for financing forest conservation activities, much of the literature on conservation finance has not clarified the effectiveness of existing results-based finance schemes [17]. Wong et al. [62] showed that while a results-based payment approach can contribute to emissions reduction, it does not guarantee an effective REDD+. An effective results-based payment approach needs to consider how results are defined and agreed upon, as well as the conditions enabling social and political acceptance [62]. In addition, moving from the readiness phase through policy design and implementation toward results-based payments for carbon and non-carbon benefits is challenging for most REDD+ countries because of numerous political–economic factors, such as political factors that are beyond the control of policy managers [60, 63]. Korhen-kurki et al. [64] showed that making REDD+ progress regardless of domestic change requires the presence of powerful transformational coalitions and strong ownership and leadership; if there were no such drivers, performance-based funding by donors could work as an incentive. Brockhaus et al.’s [60] analysis of the national political context in 13 REDD+ countries indicated that the existence of a broader policy change and the availability of performance-based funding, combined with strong national ownership of the REDD+ policy process, may help guide other countries to formulate REDD+ policies that produce efficient, effective, and equitable outcomes. There is also research on the allocation of financial resources from results-based finance schemes for REDD+ [17, 62] that links to the discussion in section “REDD+ finance aligned with the UNFCCC”.

##### National and subnational frameworks

Much of the literature regarding REDD+ finance has used case studies of recipient countries and touched on the relationship between financial issues and national and/or subnational frameworks, including institutions related to REDD+ in recipient countries. These national and/or subnational frameworks in recipient developing countries include issues on NDCs, benefit-sharing (financial allocation), safeguards, and REDD+ costs.

The NDCs of many developing countries have recognized the important role of forests, showing mitigation measures in the forest sector, including REDD+, and further developing various green initiatives to achieve their mitigation goals [65, 66]. However, these measures do not directly aim to reduce emissions, and REDD+ and NDCs may be ineffective in achieving the planned outcomes if there is a lack of clear policies and measures to address the drivers of deforestation and forest degradation, as well as a transparent monitoring and evaluation framework [65, 66]. National and subnational approaches (i.e., jurisdictional REDD+ approaches), which are based on the premise that results-based flows of finance can cause changes in complex land-use systems throughout nations or subnational jurisdictions to achieve large-scale reductions in carbon emissions from deforestation and forest degradation, are considered key elements in recipient countries to implement REDD+ on the basis of the UNFCCC [67]. However, the early jurisdictional approach experiments have shown that the promise of payments alone is insufficient to drive the transition of the jurisdictional land-use system [67]. Kissinger et al. [29] indicated that a lack of fiscal reform remains a key barrier to achieving transformative change in the land-use sector. Furthermore, the drivers of deforestation are rarely mentioned in NDCs [65].

Therefore, the development of national and subnational frameworks in recipient countries is important for REDD+. Gallo and Albrecht [68] indicated that REDD+ needs to be effectively framed as a public policy that seeks to identify those who legitimately act for forest conservation, to understand the drivers of deforestation and forest degradation, and to address deforestation drivers within the logic of integrating existing policy initiatives. Hargita et al. [69] suggested that REDD+ alone cannot foster the transformational change that is required across landscapes and sectors, and REDD+ needs to be included in other national programs that prepare the way for sustainable development.

Furthermore, the existing literature has touched on the issues of benefit-sharing and safeguards in discussions of national and subnational frameworks for REDD+. One topic that is frequently discussed regarding national and subnational frameworks is the benefit-sharing mechanism of REDD+. Discussions of benefit-sharing reflect increased concerns over how to access REDD+ finance, how to assess REDD+ policy performance and results, how to generate co-benefits, and how to resolve issues related to equity and safeguards [62]. Unclear benefit-sharing mechanisms are perceived as one element that impedes REDD+ implementation and payment distributions [70].

In realizing REDD+, there is a common understanding regarding the need to protect the rights of local communities through safeguards, as well as the recognition that they are key actors in implementing REDD+ and receiving its benefits [71, 72]. There has been a concern that the benefits derived from REDD+ will not be equitably distributed to local communities [73, 74]. Much of the literature has discussed benefit-sharing for forest-dependent local communities and increasing the prosperity of these actors [41, 75]. Early debates on benefit-sharing focused on the local level and generally addressed why, on whom, what, and how REDD+ finance should be spent and distributed at the local level [62]. Currently, developing countries use different benefit-sharing mechanisms [76].

Awung and Marchant [77] suggested that the benefit-sharing mechanism and safeguards need to be transparent and clearly designed to achieve community expectations to enable sustainable development and prevent the early failure of REDD+ projects. However, focusing on local benefit-sharing and targeting poor smallholders and communities as REDD+ beneficiaries to address equity and legitimacy may result in overlooking the larger-scale drivers that can reduce deforestation more effectively; thus, the transfer of burdens needs to be examined [62]. Several articles have shown that the current focus of REDD+ on local actors and their role in driving deforestation will act as a distraction from many other large international and national drivers of deforestation, such as large-scale industrial agriculture [72, 78].

Another issue related to benefit-sharing and safeguarding recipients is corruption [19]. Corruption is a major challenge in the effective implementation of REDD+ programs and thus needs to be addressed. Developing a fair and transparent benefit-sharing mechanism is one way to avoid corruption [19]. However, regarding REDD+ safeguards, one study showed that, at best, they are likely to have only a partial effect on corruption, and at worst, not reduce corruption at all [79].

Furthermore, there are issues regarding costs. Concerns have repeatedly been raised about whether actors in REDD+ host countries will end up bearing the costs of REDD+ [80]. Currently, many organizations involved in REDD+ implementation, especially at the subnational level and in the public sector, are bearing implementation costs that are not covered by the budgets of REDD+ initiatives [80]. Previous studies on REDD+ costs have focused on the opportunity costs of foregoing alternative uses of forest land, such as the conversion of forest into cropland, and it is important to consider ways to provide incentives for actors in recipient countries to implement REDD+ [81, 82]. While accounting for the fact that the forest reference emission levels/forest reference levels and MRV are the basis for financing these REDD+ activities [9, 83], considering sustainable cost-sharing of implementation costs is also an important element [80].

#### REDD+-related finance outside the UNFCCC

##### Incentives for private sector participation

As described in section “REDD+ finance aligned with the UNFCCC”, many developing countries have incorporated REDD+ into their NDCs as their contribution to climate change; however, this is not the case for most developed countries [65, 84]. This indicates that developed countries do not consider international support for REDD+ as part of domestic contribution, or they consider that REDD+ is a voluntary commitment outside the UNFCCC, rather than an attractive mechanism for private investors to reduce carbon emissions [65, 84]. Therefore, it is important to examine the incentive design in REDD+ to raise more private finances and to encourage more countries to incorporate REDD+ into their NDCs [84]. Because of the current inadequate public finances, a wide range of actors, including the parties to the UNFCCC, are turning toward the private sector to scale up REDD+ finance [48]. As mentioned above, current private sector contributions to REDD+ mainly occur through voluntary carbon markets and the project-scale payments for carbon offsets/units [27, 44].

Private sector engagement in REDD+ finance is limited for various reasons, which have been identified in the academic literature [4, 15, 41, 85–87]. The challenges include the need for more certainty in climate policy and markets and a clear regulatory framework [4, 85–87]; strong forest governance (e.g., land tenure reform, land-use planning, and the strengthening of law enforcement and forest institutions); clear understanding of carbon rights (which are justified claims that there is a benefit from reduced GHG emissions and/or carbon sequestration, and these justifications can be based on an activity that leads to forest conservation or an asset) and transparent regulation on who can benefit from national REDD+ [41]; and the implementation of REDD+ activities at different national and subnational levels [15].

To increase private finance inputs into REDD+, the importance of understanding the rationale, strategies, and behavior of private sector actors has been recognized [85]. Although this remains an under-researched field, existing studies have focused on the short-term behavior of actors in REDD+ [85, 88]. The benefit and opportunity costs associated with REDD+ implementation have also been an important research topic in REDD+ finance because they enable private investors to evaluate the returns (i.e., carbon offsets) from their investments (i.e., purchasing carbon credits) from a business aspect [89]. One issue related to the opportunity costs associated with REDD+ is the relatively modest profits gained from forest carbon financing compared to those gained from oil palm and timber plantations, meaning that REDD+ has difficulty slowing these lucrative industries [90]. In addition, there are challenges from the viewpoint of recipients who provide investment opportunities. For example, investment opportunities on the ground may be unable to meet the financial requirements of commercial investors, especially when the costs of technical assistance, monitoring, and enforcing environmental standards increase [91]. Sheng [88] showed that incentive–coordination contracts among actors in REDD+, whereby private investors and landholders cooperate to coordinate their benefits, can improve private investors’ understanding of the value and risks associated with REDD+ projects and can attract private sector participation in REDD+.

In addition to the characteristics of private sector actors and their incentives for participating in REDD+, there are challenges associated with the nature of REDD+ as a natural climate solution, including the costs and complexity of the MRV of REDD+ activities. These challenges are attributed to the need to consider the risks of permanence (whether sequestered carbon will remain in the forest) and carbon leakage (whether deforestation will occur elsewhere because of the projects), as well as to precisely determine and monitor forest carbon sinks [12, 57]. These issues may affect the incentives for private sector participation.

##### Private carbon finance and voluntary carbon markets

The uncertainty of compliance carbon markets has also been a challenge for private-sector involvement in REDD+ finance. At the international level, integrating climate cooperation through carbon markets into Article 6 of the Paris Agreement and including REDD+ may enable more cost-effective emission reductions [26, 92]. At the national and subnational levels, compliance carbon markets, such as those in New Zealand, Australia, Colombia, and California, accept forest carbon units, but how the compliance carbon markets led by national and subnational governments will deal with REDD+ remains uncertain [15, 26, 41].

Voluntary carbon markets have also enhanced private sector participation in REDD+ finance. The private sector’s enthusiasm for voluntary carbon markets and nature-based carbon credits could be targeted toward investments in REDD+ projects (to generate carbon credits because of their interests in offsets) and could be targeted toward corporate supply chain efforts designed to deliver deforestation-free commodities [93].

The number of voluntary projects marketing offsets to buyers motivated by corporate social responsibility or in expectation of future compliance obligations has continued to increase [94]. The total value of voluntary carbon markets in 2020 (USD 473 million) was the highest annual value observed since 2012, with market transactions exceeding USD 748 million as of August 2021 [42]. Forestry and land use, a leading category in the volumes transacted, set a near record in 2020 at 47 MtCO_2_e with a peak price of $5.59/ton in 2020 [42]. REDD+-related volumes of forestry and land-use project types from 2020 to 2021 grew dramatically, including a 166% increase in the avoided unplanned deforestation project type and a 972% increase in avoided planned deforestation [42].

One challenge related to voluntary carbon markets is that their carbon price has been low [70], which could reduce the incentives for the private sector to implement REDD+. Although further analysis is required in this respect, REDD+-related volumes have substantially increased, as has voluntary carbon markets’ issuance of forestry and land use projects, which grew from approximately USD 57.2 million in 2020 to USD 107.5 million in 2021 [42].

Another issue is the relationship between projects and national-level carbon accounting because gaps between them may affect the leakage problem described in section “REDD+-related finance outside the UNFCCC” [93, 95]. West et al. [95] showed that, although there have been efforts to integrate the reduced carbon emissions from deforestation through voluntary REDD+ projects into national GHG emission inventories, credible evidence on the effectiveness of these voluntary activities is limited. However, Streck [93] reported that nesting different activity levels (national, subnational, and project) and accounting would allow public and private sector interventions to become part of policy-integrated jurisdictional programs.

Furthermore, there are concerns that voluntary carbon markets could lead to company greenwashing (when companies try to make it appear that they are contributing more to the environment than they actually are) and undermining the goals of the Paris Agreement [96, 97]. These greenwashing challenges can be mitigated by considering robust standards and rules, having increased transparency from both voluntary carbon market operators and credit buyers, and having public reporting of GHG accounting and receipts that correlate the source of the credits with mitigated emissions [96, 97].

##### New investment and finance opportunities

Because of insufficient finance for REDD+, new investment and finance opportunities and models for REDD+ are being investigated [15]. The exploration of new finance opportunities for the forest sector, including REDD+, incorporates new blended finance models that combine different finance sources, such as public and private finance [22, 91], as well as enhanced bonds for forest-based mitigation activities [98]. Rode et al. [91] found that blended finance models that combine funding from commercial, public, and philanthropic sources could contribute to financing sustainable landscapes. They also showed that philanthropic sources would cover the costs of securing direct conservation benefits or monitoring environmental impacts, and non-governmental organizations or stakeholders would provide technical assistance to help implement the transition at the farm level [91]. Furthermore, governments or international development banks would provide “de-risking” components, and private commercial impact investors would receive the same financial returns as traditional investments [91].

Golub et al. [99] discussed how bonds and put options can help bring upfront investment to stimulate REDD+ supply. They showed that bonds backed by public or private funding can provide upfront resources for jurisdictions to develop and strengthen REDD+ programs, including forest conservation and agricultural intensification, and to gain commitments from investors [99]. Bracking et al. [100] showed that, as for green bonds, “REDD+, the GCF, and green bonds are already correlated” in the UNFCCC documents, particularly following the Warsaw Agreement in 2013 and the Paris Agreement in 2015. In addition, the search for product commensurability has begun to create aggregated canopy products (which could contain various forms of climate finance components, such as green bonds, payments for ecosystem services schemes, REDD+ projects, or groups of certified emissions reductions) [100].

Although the literature on new finance opportunities for REDD+ is limited, they have been discussed in the context of nature-based solutions (NbS), which comprise a wide range of measures, including REDD+. NbS are “actions to protect, sustainably manage and restore natural or modified ecosystems that address societal challenges effectively and adaptively, simultaneously providing human well-being and biodiversity benefits” [101]. The academic literature sorted by the keyword “REDD+ financ*” contains limited discussion on NbS; however, some recent studies have considered NbS while incorporating REDD+ discussions [93, 102, 103]. Although the development of NbS faces barriers related to the value proposition, value delivery, and value capture of NbS business models and available sustainable public/private funding sources, there is an increasing need to establish new finance approaches and business models to attract both public and private finance to NbS [15, 104–107].

There is also growing recognition from financial institutions and companies regarding the need to address not only the risks associated with climate change but also those associated with declining biodiversity, which include physical, transition, and reputational risks [108–110]. An increasing level of research that enables us to understand and identify nature-related financial risks [108, 111, 112] could contribute to promoting more investment and finance in NbS, including REDD+.

### Utilizing challenges and lessons learned to improve REDD+ finance and broader environmental finance

On the basis of the challenges and lessons learned for REDD+ finance and its governance from the literature review, in this section, we discuss ways to overcome these challenges.

#### Findings from literature review

The key findings of this review are summarized as follows.

1. Coordination of multilateral and bilateral financing (section “REDD+ finance aligned with the UNFCCC”)
  The fragmentation and complexity of REDD+ finance still occurs because it comprises different financing sources, mechanisms, and programs/projects, as well as different relationships between funders and recipients and the consideration of recipient domestic situations.
  The GCF and SCF can only partially address the fragmentation and complexity of REDD+ finance.
2. Results-based finance (section “REDD+ finance aligned with the UNFCCC”)
  REDD+ results-based finance approaches vary with different financing sources (non-market-and market-based finance) and targets (national and/or subnational programs aligned with the UNFCCC or projects that are mainly implemented outside the UNFCCC).
  The challenges and lessons learned from REDD+ results-based finance, such as multilateral funds, the GCF, and the Amazon Fund, include the difficulty of the GCF in mobilizing additional finance for REDD+ due to its institutional constraints, the donors placing institutional burden on recipient countries’ ability to access finance, and a lack of targeted funding strategies to maximize the fund results.
  Studies on REDD+ performance, where results-based finance is the core of the discussion, have increased. The effectiveness of results-based finance schemes depends on the way results are defined and the conditions that enable social and political acceptance, including political–economic factors in recipient countries. In addition, it is important to have performance-based funding in combination with strong national ownership of the REDD+ process.
3. National and subnational frameworks (section “REDD+ finance aligned with the UNFCCC”)
  The NDCs of many developing countries show mitigation measures in the forest sector. To tailor these measures to emissions reduction, clear policies and measures are required to address the drivers of deforestation and forest degradation, as well as transparent monitoring and evaluation frameworks. For this purpose, national and subnational approaches (jurisdictional approaches) could enhance changes to complex land-use systems so that they can achieve large-scale emissions reduction.
  Other challenges that are being studied regarding national and subnational frameworks include improving benefit-sharing mechanisms and safeguards, especially in terms of distribution to local communities. However, it is also important not to overlook the larger-scale drivers of deforestation. Furthermore, corruption among recipients and the sharing of REDD+ implementation costs also need to be addressed at the national and subnational levels.
4. Incentives for private sector participation (section “REDD+-related finance outside the UNFCCC”)
  Although it is important to examine ways to incentivize the private sector, various factors limit private-sector engagement in REDD+ finance. These factors include uncertainty over climate policy and markets, weak forest governance, and a lack of clear understanding of carbon rights.
  There is a growing need to understand the rationale, strategies, and behavior of private sector actors, which are linked to the opportunity costs associated with REDD+.
  Other challenges are associated with the nature of REDD+ as a natural climate solution, the costs and complexity of MRV for REDD+ activities because of the need to address the risks of permanence and carbon leakage, and the monitoring of carbon sinks.
5. Private carbon finance and voluntary carbon markets (section “REDD+-related finance outside the UNFCCC”)
  The uncertainty of compliance carbon markets has also been a challenge for the involvement of the private sector in REDD+ finance, although voluntary carbon markets have enhanced private sector participation.
  Forestry and land use is the leading category of volumes transacted in voluntary carbon markets, while the challenges of these markets include the low carbon price, the relationship between projects and national-level accounting (nesting activities and accounting at different levels can make the public and private interventions part of policy-integrated jurisdictional programs), and company greenwashing.
6. New investment and finance opportunities (section “REDD+-related finance outside the UNFCCC”)
  Studies have explored new finance opportunities and models for REDD+, such as building new blended finance models (which combine public and private finance, including philanthropic sources, and different actors’ finance and actions can complement each other) and developing enhanced bonds for mitigation in the forest sector (e.g., REDD+, the GCF, and green bonds are interrelated).
  There is growing discussion on establishing new finance opportunities and business models for NbS, including REDD+, and on the needs for financial institutions and companies to address climate change and nature-related risks.

#### Improving REDD+ finance and its governance

The literature review indicated that the two streams of discussion on REDD+ finance—those aligned with and those outside the UNFCCC—have developed differently and have different challenges. The discussion on REDD+ finance aligned with the UNFCCC has focused on enhancing the performance of REDD+ finance using mainly public finance, including results-based finance and the jurisdictional approach. In contrast, the discussion of REDD+-related finance outside the UNFCCC has focused on ways to enhance the engagement of the private sector in REDD+ finance, mainly targeting the project level. The discussion includes the relationship between voluntary carbon markets and other investment and finance mechanisms.

Many existing studies have not comprehensively discussed the two streams of REDD+ finance and its governance, and the term REDD+ has been used differently in the literature. However, through this literature review, we identified common governance challenges in the two streams. One challenge is the need to consider REDD+ finance in terms of linkages with other targets and policies, such as net-zero/carbon neutrality, deforestation-free supply chains, and NbS [31, 93, 113–115]. Various objectives and policies can enhance the performance of REDD+, although coordination is required among relevant institutions and actors (such as national and subnational governments in developing countries, donors, companies, financial institutions, and non-governmental organizations) at different levels, which are international, national, and local. With regard to net-zero/carbon neutrality, many countries and regions, such as the European Union, China, Japan, South Korea, Canada and the United States pledged net-zero commitments. Such pledges were also made by many non-state actors, including the private sector and local governments. Furthermore, the potential use of forests as carbon sinks has received increasing attention. Regarding deforestation-free supply chain initiatives, an increasing number of companies have announced zero-deforestation commitments to eliminate commodities produced at the expense of forests from their supply chains [116]. Hargita et al. [69] showed that the linkage between REDD+ and deforestation-free supply chain initiatives provides many complementarities that could foster the goal of halting deforestation. Linking the initiatives could address the criticism that REDD+ overlooks the large drivers of deforestation, as discussed in section “REDD+ finance aligned with the UNFCCC”.

As described in section “REDD+-related finance outside the UNFCCC”, investment and finance have been explored in the context of NbS, including REDD+. Although the concept of NbS was not developed within the UNFCCC [5], the UNFCCC SCF organized the SCF Forum on Finance for Nature-based Solutions. Part I of the forum took place in 2021 and Part II took place in 2022. In this forum, the ways to mobilize and deliver public and private finance for NbS—as well as the challenges of existing financial instruments (e.g., blended finance, insurance, microfinancing, and nature bonds), multilateral and bilateral support, and other international institutions—were discussed [51, 117]. These discussions indicated that NbS are already well recognized within the UNFCCC, and more interaction between the two discourses of investment and finance for REDD+ and NbS is necessary.

Another issue is the need to develop learning systems and techniques for REDD+ finance [18, 54, 75, 76, 118]. The pressure to learn from efforts, including REDD+, continues to grow because of the global urgency to reduce GHG emissions [64]. Pinsky et al. [118] highlighted the importance of determining whether REDD+-related funds actually generate experimental learning and policy improvement for reforming REDD+ incentive schemes. Schroeder et al. [18] were one of the researchers who examined learning in REDD+ finance. They explored learning for REDD+ and the REDD+ funding landscapes in Norway, Germany, and the United Kingdom and showed that the historical, institutional, organizational, operational, and political approaches of the three countries vary and have generated different lessons. Such discussions and studies conducted to develop learning systems and techniques for REDD+ finance are essential to develop REDD+ finance as well as the broader environmental finance.

## Conclusions

We reviewed the literature on the challenges and lessons learned for REDD+ finance and its governance in two areas: (1) REDD+ finance aligned with the UNFCCC and (2) REDD+-related finance outside the UNFCCC. We identified six key elements for REDD+ finance and its governance: (1) coordination of multilateral and bilateral financing, (2) results-based finance, (3) national and subnational frameworks, (4) incentives for private sector participation, (5) private carbon finance and voluntary carbon markets, and (6) new investment and finance opportunities. Based on these key elements, we reviewed the challenges and lessons learned for REDD+ finance and its governance aligned with and outside the UNFCCC.

The discussion on REDD+ finance aligned with the UNFCCC has focused on enhancing the performance of REDD+ finance using mainly public finance and includes results-based finance and the jurisdictional approach. In contrast, the discussion of REDD+-related finance outside the UNFCCC has focused on ways to enhance the engagement of the private sector, mainly targeting the project level, and includes the relationship between voluntary carbon markets and other investment and finance mechanisms.

Although there is a lack of comprehensive discussion of the above two streams, there are common governance challenges. One is the need to consider REDD+ finance in terms of linkages with other objectives and policies, such as net-zero/carbon neutrality, deforestation-free supply chains, and NbS. Another is the need to develop learning systems and techniques for REDD+ finance. These areas need to be explored to improve REDD+ finance as well as the broader environmental finance.

## Data Availability

Not applicable. This paper reviewed literature, mainly academic literature.

## Abbreviations

CAFI: Central African Forest Initiative
CBFF: Congo Basin Forest Fund
CF: Carbon Fund
COP: Conference of the Parties
CO_2_: Carbon dioxide
FCPF: Forest Carbon Partnership Facility
FIP: Forest Investment Program
GCF: Green Climate Fund
GEF SFM: Global Environment Facility Sustainable Forest Management Programme
GHG: Greenhouse gas
ISFL: Initiative for Sustainable Forest Landscapes
MRV: Measurement, reporting, and verification
NbS: Nature-based solutions
NDCs: Nationally determined contributions
RF: Readiness Fund
REDD+: Reducing emissions from deforestation and forest degradation, and the role of conservation, sustainable management of forests and enhancement of forest carbon stocks in developing countries
SCF: Standing Committee on Finance
UNFCCC: United Nations Framework Convention on Climate Change
